# Cannabinoids and Reproduction: A Lasting and Intriguing History

**DOI:** 10.3390/ph3103275

**Published:** 2010-10-25

**Authors:** Giovanna Cacciola, Rosanna Chianese, Teresa Chioccarelli, Vincenza Ciaramella, Silvia Fasano, Riccardo Pierantoni, Rosaria Meccariello, Gilda Cobellis

**Affiliations:** 1 Dipartimento di Medicina Sperimentale, Sez. “F. Bottazzi”, Seconda Università degli Studi di Napoli, Napoli, Italy; 2 Dipartimento di Studi delle Istituzioni e dei Sistemi Territoriali, Università di Napoli “Parthenope”, Napoli, Italy

**Keywords:** cannabinergic system, male reproduction, female reproduction, hypothalamus-pituitary-gonads axis

## Abstract

Starting from an historical overview of lasting *Cannabis* use over the centuries, we will focus on a description of the cannabinergic system, with a comprehensive analysis of chemical and pharmacological properties of endogenous and synthetic cannabimimetic analogues. The metabolic pathways and the signal transduction mechanisms, activated by cannabinoid receptors stimulation, will also be discussed. In particular, we will point out the action of cannabinoids and endocannabinoids on the different neuronal networks involved in reproductive axis, and locally, on male and female reproductive tracts, by emphasizing the pivotal role played by this system in the control of fertility.

## Contents

1. The Cannabinergic System: A Historical Overview
  1.1.1 Cannabinoid receptors
  1.1.2 Exogenous and endogenous ligands
  1.1.3 Endocannabinoids biosynthesis and degradation
2. Interactions of the Cannabinergic System with Different Neuronal Networks in Reproductive Perspective
3. The Cannabinergic System in Male Reproductive Tracts: From Spermatogenesis to Sperm Physiology
  3.1.1 Testis
  3.1.2 Excurrent duct system
4. Effects of the Cannabinergic Sytem on Female Reproduction: From Ovary to Utero-placental Relationship
5. Closing Remarks

## 1. The Cannabinergic System: A Historical Overview

*Cannabis sativa* (or marijuana) is one of the oldest psychotropic drugs known to humans. According to archaeological discoveries, its use was already mentioned in the Pen Ts'ao, a Chinese pharmacopeia, around 4,000 BC, where it is reported that *“Cannabis is spicy when eaten but has a poison good for the five organs. It helps much your energy, your whole body, stops sweat (because of cold) and leaves the water from the body, urine”* [1]. Moreover, the same book reports the first description of the hallucinogenic effects of the plant: “*If you eat more, you will see white ghosts walking around and if you eat long enough, you will know how to talk to the Gods*”. However, is is hard to precisely date early *Cannabis* use because the oral traditions only began to be written starting from 2,737 BC. In that year, the Chinese emperor Shen Nung was the first to describe the properties of *Cannabis* in his compendium of medical herbs [2]. Afterwards, around 1,400 BC, *Cannabis,* named Bhang, was reported in the Indian holy book *Atharvaveda* in relation to practices against diseases and demons [3].

Subsequent fine descriptions of *Cannabis* can be found in Egyptian, Greek and Latin books. Around 70 AC Dioscorides, a surgeon in the Roman legions under the Emperor Nero, provided in his herbarium *De materia medica*, (cap CLXV, book III) a precise description of *Cannabis* and suggested its therapeutical use in case of earache (“*Ex eo recente expressus succus convenienter aurium doloribus instillatur*”). Additionally, he also described *Cannabis sylvestri* (also known as *Cannabis indica*) and indicated its beneficial effect in case of inflammation, oedema and gout (“*Cocta autem et imposita radix vim habet inflammations leniendi, oedemata discutiendi et articulorum tophos dissipandi*”). *Cannabis* medical use diffused worldwide even in the New World, to where hemp cultivation was exported by the Spanish Conquistadores to provide ropes and clothes [2]. In Southern Europe, medical interest in *Cannabis* was awakened by Napoleon's campaign in Egypt, when the health effects were observed among soldiers [4]. 

In 1839, William O'Shaughnessy, a British physician and surgeon working in India, was the first to describe the analgesic, muscle relaxant and anticonvulsant properties of *Cannabis*. His observations quickly led to the expansion of the medical use of *Cannabis*. Indeed, it was even prescribed to Queen Victoria for relief of dysmenorrhea [5], although this seems the only therapeutical benefit described in female reproductive system.

In the USA, in 1854 the United States Dispensatory included *Cannabis*, which was sold freely in pharmacies of Western countries [6]. However, during the “Noble Experiment” (1920-1933), when sale, manufacture and transportation of alcohol for consumption were banned nationally, the American authorities condemned the use of *Cannabis*, making it responsible for moral and intellectual deterioration and violence. Thus, in 1937, Marijuana Tax Act made possession or transfer of *Cannabis* illegal throughout the United States under federal law [7]. Additionally, in 1942, *Cannabis* was removed from the United States Pharmacopeia, thus losing its therapeutic legitimacy [8]. 

Today, the long lasting use of *Cannabis* accross the centuries is not a warranty of its therapeutical efficacy. For example, mandrake and cantaris, two famous remedies, are completely abandoned nowadays because of their side effects [9], so caution should be used before accepting any old drug as a therapeutical agent simply based on its lasting therapeutical history. In order to evaluate the safety and efficacy of *Cannabis*, investigations into the chemestry of *Cannabis* and identification of its active components can be traced back to the 19^th^ century. At the beginning of this reserch, an alkaloid was considered the active constituent of *Cannabis*. Only in 1965, Mechoulam and Gaoni determined the correct chemical structure of Δ-9-tetrahydrocannabinol, commonly known as THC, the major psychoactive ingredient of *Cannabis* [10]. From this starting point, intensive research was carried out to identify the other components of *Cannabis*, leading to the identification of a total of 483 constituents [11]. Other cannabinoids (CBs) present in Indian hemp include Δ-8-tetrahydrocannabinol (Δ^8^-THC), cannabinol (CBN), cannabidiol (CBD), cannabicyclol (CBL), cannabichromene (CBC) and cannabigerol (CBG), but they are present in small quantities and have no significant psychotropic effects compared to THC. However, they may have an impact on the product's overall effect [12].

In 1987, new potent cannabinoid agonists were developed. This group of CBs consists of ABC-tricyclic dibenzopyran derivatives, as 11- hydroxy-Δ^8^-THC-dimethylheptyl (HU-210) and desacetyl-l-nantradol. These CBs elicited cannabimimetic responses both *in vivo* and *in vitro* [13].

### 1.1. Cannabinoid receptors

Due to the lipophilic nature of CBs, an intracellular receptor was suspected. However, in 1988, the identification of a high affinity, stereoselective, pharmacologically distinct cannabinoid receptor (CBR) on the plasma membrane (PM) in brain tissue was reported [14]. Therefore, it was suggested that CBs exert their actions by binding to specific membrane receptors. The CB_1_ (cannabinoid receptor type 1), cloned by Matsuda in 1990 [15], and the CB_2_ (cannabinoid receptor type 2), identified by Munro in 1993 [16], are members of the superfamily of seven transmembrane (TM)-domains GTP-binding protein-coupled receptors (GPCR) [17]. They share 44% overall identity, especially in the 2, 3, 5 and 6 TM regions, with CB_1_ being larger than CB_2_ at N-terminal, third extracellular loop and C-terminal regions level [18]. Whereas CBs interact with CB_1_ in the pore formed within the TM helical cluster, the three cytosolic loops contribute to the activation of G proteins [19]. With respect to CB_1_, several downstream signal transduction pathways have been characterised. First of all, CB_1_ interaction with G_i/o_ proteins - pharmacologically blocked by the treatment with pertussis toxin - inhibits adenylyl cyclase (AC), with the consequent decrease of cAMP levels [20]. CB_1_ can also be coupled to G_s_ instead of G_i_ and elicit cAMP accumulation, but the physiological significance of this duality needs more investigation [21]. Moreover, CBs inhibit voltage-gated L, N, and P/Q-type Ca^2+^ channels and stimulate K^+^ channels through CB_1_ activation [22,23,24] or an indefinite receptor-indipendent- mechanism [25]. CB_1_ coupling to G_q/11_ proteins - with consequent phospholipase C (PLC) and constitutive nitric oxide synthase (cNOS) activation - also mediates the increase in intracellular Ca^2+^, probably due to release of this ion from intracellular stores [26,27,28]. Finally, mitogen-activated protein (MAP) kinase pathway is positively regulated by CB_1_ [29]. The possibility that MAP kinase stimulation is independent by CB_1_ activation, but is influenced by cAMP levels decrease, has also been explored [30]. 

CB_2_ primarily acts through G_i/o_ proteins [31] with consequent activation of p42/44 MAP kinase, extracellular signal-regulated protein kinases (ERK) and induction of gene expression through protein kinase C (PKC) stimulation [32]. 

CB_1_ is primarily, but not exclusively, expressed in the central nervous system (CNS) and mediates the central CBs effects. Peripherally, CB_1_ expression has been found in the pituitary gland [33], immune cells [34,35], reproductive [36] and gastrointestinal tissues [37], superior cervical ganglion [38], blood vessels [39], lung [40], bladder [41], adrenal gland [42], liver [43] and adipose tissue [44].

CB_2_ expression, on the other hand, was restricted for a long time only to the periphery, mainly in immune cells (B and natural killer cells) [45], spleen [16], thymus [45], tonsils [46], splenic macrophage/monocyte preparations [45], mast cells [47], and peripheral blood leukocytes [35]. Recently, it has been reported that CB_2_ expression in neuronal microglia cells [48], brain stem cells [49], cerebellum, striatum, midbrain and hippocampus [50].

Pharmacological evidences exist for the presence of other CBRs, not yet cloned, that work differently by CB_1_ and CB_2_, as G protein-coupled receptor 55 and 119 (GPR55 and GPR119) [51,52] and transient potential vanilloid channel type 1 (TRPV1). 

GPR55 has been considered a new cannabinoid receptor [53], probably also activated by endogenous l-α-lysophosphatidylinositol (LPI) [54]. The activation of GPR55 by CBs induces Ca^2+^ release from intracellular stores, *via* G_q_ and PLC [55]. GPR55 has also been reported to couple to G*_α_*_12_ and activate RhoA [55]. Interestingly, Lauckner *et al.* [53] did not demonstrate any activation of ERK1/2 kinase pathway in response to GPR55 stimulation. Therefore, the effects of LPI have been associated with ERK activation and a modest increase in cytosolic Ca^2+^, both pathways involving G*_α_*_12_/G*_α_*_13_ and RhoA [53]. Lastly, Kapur *et al.* [56] have suggested that LPI, SR141716A and AM251 work as GPR55 agonists. These findings undoubtedly indicate that GPR55 should at best be classified as an atypical cannabinoid receptor. Recently, high *gpr55* mRNA levels have been detected in murine adrenals, gastrointestinal tract and CNS [57], whereas GPR55 protein has been localised in mouse arteries [58].

GPR119 is an orphan receptor identified through a bioinformatics approach [52], predominantly expressed in pancreatic and intestinal tissues [59]. GPR119 behaves as a Gs coupled receptor, in fact specific agonists stimulate adenylyl cyclase in cells transfected with this receptor [60]. The identification of GPR119 as a hypothetical CBR derives from some evidences reporting the activation of this receptor by *N*-oleoylethanolamine (OEA), *N*-palmitoylethanolamine (PEA) and AEA. However AEA displays very weak effect [60].

TRPV1 - a six-domain-TM nonselective cation channel - exists as a homomeric or heteromeric complex composed of four subunits that assemble to form functional cation-permeable pores, usually localised in the PM, with intracellular N and C terminals [61]. It is activated by a wide variety of physical (temperature, light, pH, mechanical pressure, *etc*) and chemical (acids, alkali, endogenous lipids, *etc*) stimuli; the best-known activators of this channel are temperatures greater than 43°C and capsaicin (CPS), the pungent compound found in hot peppers [62]. However, it is also known to have voltage-dependent gating properties thus to allow the passage of currents through PM [63]. Like many other channels, TRPV1 contains multiple phosphorylation sites in its amino acid sequence for PKC [64] and protein kinase A (PKA) [65], implicating that its activity is strongly influenced by these kinases. TRPV1 has been immunolocalised in rat larynx [66], trigeminal ganglion [67], and mammalian male germ cells [68,69], as it will be further described. 

### 1.2. Exogenous and endogenous ligands

Cannabinoids can be divided into different groups: classical and non classical CBs, aminoalkyl-indoles and eicosanoids [17]. The first group consists of ABC-tricyclic dibenzopyran derivatives that are either compounds occurring naturally in the plant, *C. sativa*, or its synthetic analogs. The most investigated among classical CBs are Δ^9^-THC, Δ^8^-THC, HU-210 and desacetyl-l-nantradol. They bind CB_1_ and CB_2_ without major selectivity for either of these receptors [17]. The non classical CBs group includes AC-bicyclic and ACD-tricyclic cannabinoid analogs, lacking the dihydropyran ring of THC, such as CP55940, which represents the prototypical compound of this series [13]. Aminoalkylindoles are cannabimimetic compounds not structurally derived from THC, such as *R*-(+)-WIN55212, which displays high affinity for both CBRs, with moderate selectivity in favor of CB_2_ [70]. Finally, eicosanoids are endogenous fatty acid amides (endocannabinoids, eCBs). Anandamide (AEA) was the first endocannabinoid isolated in mammalian brain [71], followed by other compounds as 2-arachidonoylglycerol (2-AG) [72], 2-arachidonoylglyceril ether (noladin, 2-AGE) [73], virodhamine [74] and *N*-arachidonoyldopamine (NADA) [75]. Among these eCBs, the most investigated to date have been AEA and 2-AG. Furthermore, eCBs, as lipophilic molecules, are not stored in vesicles, and exist as integral constituents of PM, from which they are synthesised 'on demand' [76]. Evidences indicate that AEA can activate rat or human TRPV1 in transfected cells to produce membrane currents or increase intracellular Ca^2+^ [77]. 

“Endocannabinoid-like” molecules, as OEA and PEA, however, are able to activate unexpected molecular targets like TRPV1, peroxisome proliferator activator receptor alpha (PPARα) [78]. Finally, CBRs antagonists include diarylpyrazoles, where the prototypic members are SR141716A (or Rimonabant) and SR144528, potent CB_1_ and CB_2_-selective ligands, respectively [79,80]. Diarylpyrazoles can behave also as “inverse agonists”, which reduce the constitutive activity of CBRs in the absence of ligands [81]. For this reason, analogs of SR141716A, as AM251 and AM281 [82], or SR144528, as AM630 [83] were developed to block CB_1_ and CB_2_-mediated effects, respectively (the steps of the cannabinoid research-discoveries and a summarising list of CBs/eCBs have been summarized in Table 1, Table 2).

**Table 1:** List of cannabinoid research discoveries.

Discovery	Ref.
Δ^9^-THC	[10]
CB_1_ receptor	[15]
CB_2_ receptor	[16]
TRPV1	[61]
GPR119	[52]
GPR55	[51]
AEA	[71]
2-AG	[72]
SR141716A	[79]
SR144528	[80]
AM251 and/or AM281	[82]
AM630	[83]

**Table 2:** List of CBs/eCBs.

*Classical cannabinoids*
Δ-9-tetrahydrocannabinol, Δ^9^-THC [10, 17]
Δ-8-tetrahydrocannabinol, Δ^8^-THC [12, 17]
Cannabinol, CBN [12, 17]
Cannabidiol, CBD [12, 17]
Cannabicyclol, CBL [12, 17]
Cannabichromene, CBC [12, 17]
Cannabigerol, CBG [12, 17]
HU-210 [13, 17]
Desacetyl-l-nantradol [13, 17]
***Non classical cannabinoids***
CP55940 [70]
*R*-(+)-WIN55212 [70]
***Endocannabinoids***
AEA [71]
2-AG [72]
noladin, 2-AGE [73]
virodhamine [74]
*N*-arachidonoyldopamine (NADA) [75]

### 1.3. Endocannabinoids biosynthesis and degradation

Different pathways are involved in eCBs synthesis and release. AEA is generated from *N*-arachidonylphosphatidylethanolamine (N-ArPE) [84], which derives from the transfer of arachidonic acid from the sn-1 position of 1,2-*sn*-diarachidonylphosphatidylcholine (PC) to phosphatidyl-ethanolamine (PE), through a reaction catalysed by a calcium-dependent *N*-acyltransacylase (NAT) [85,86]. The second step is the hydrolysis of N-ArPE by an *N-*acylphosphatidylethanolamine (NAPE)-specific phospholipase D (PLD) (NAPE-PLD) [87], a member of the Ca^2+^ sensitive metallolactamase family, which releases AEA and phosphatidic acid [88]. However, since NAPE-PLD knock-out mice (NAPE-PLD KO) show unaltered brain polyunsaturated *N*-acylethanolamine (NAE) levels with [89], alternative pathways for AEA synthesis possibly exist. First, a secretory phospholipase 2 (sPLA_2_) hydrolyses NArPE to *N*-arachidonoyl-lysophosphatidylethanolamine (lyso-NArPE), which, in turn, is converted in AEA, *via* a selective lyso phospholipase D (lyso-PLD) [90]. Alternatively, from NArPE cleavage by phospholipase C (PLC), phospho-AEA (p-AEA) is formed and then dephosphorylated by a protein tyrosine phosphatase [91]. Lastly, NArPE can be deacylated by a lyso-phospholipase/ phospholipase B, generating thus glycero-phospho-AEA (glycero-p-AEA) that is cleaved to AEA by a phosphodiesterase [92]. 

The 2-AG biosynthesis occurs through a two steps mechanism: phosphitidylinositol-specific phospolipase C (PI-PLC) produces 1,2-diacylglycerol (1,2-DAG) from phosphatidylinositol (PI) [93]. Afterwards, DAGs are converted to 2-AG by diacylglycerol lipase (DAGL) [94]. In a second pathway, phospholipase A_1_ (PLA_1_) hydrolyses PI producing LPI, which is converted in 2-AG by a specific lyso-PLC [95]. Recently, two *dagl* isoforms, α and β, have been cloned from rat brain [96]. These enzymes are localised in PM and are members of the serine-lipase family with serine and aspartic acid participating in the catalytic triad [96]. 

Once produced, eCBs, as autocrine or paracrine mediators, are released in the extracellular space to bind and activate CBRs by a passive or facilitated diffusion/endocytosis across PM [84,97]. Due to its hydrophobic nature, AEA can traverse the PM [98], suggesting that AEA uptake occurs by passive diffusion [98,99]. However it is also possible a facilitated diffusion mediated by an hypothetical, not cloned yet, carrier protein named “eCBs membrane transporter” (EMT) [100], since cellular uptake follows a saturable, temperature-dependent course [101] and can be blocked by synthetic inhibitors such as *N*-(4-hydroxyphenyl)-arachidonylamide (AM404) [97]. Recently, using nanotechnologies and TRPV1 as a biosensor, the idea that AEA uptake is facilitated by a specific carrier, possibly in concert with intracellular trafficking proteins, has been strengthened [102].

Recent studies have shed light on the involvement of lipid rafts in the CBs signalling. They are sub-domains of the PM with high concentrations of cholesterol and glycosphingolipids [103]. Bari *et al.* [104] substantiated the notion that caveolae, flask-shaped membrane invaginations rich in caveolins [105], are the main membranous sites of CB_1_. Interestingly, Kaczocha *et al*. [106] have postulated the existence of AEA intracellular carriers. They belong to fatty acid binding proteins (FABP) family, which includes FABP3, FABP5 and FABP7, widely expressed in the brain. In addition to fatty acids, these proteins seem to carry other lipophilic ligands such as retinoic acid [107].

Once completed their biological activity, eCBs are inactivated by a mechanism of cellular reuptake followed by an intracellular degradation performed by hydrolytic enzymes. AEA is metabolised by fatty acid amide hydrolase (FAAH) [108] and 2-AG by monoacylglycerol lipase (MAGL) and to a lesser extent by FAAH [109]. 

AEA and 2-AG can be also susceptible to oxidative mechanisms catalysed by lipoxygenases (LOXs) and cyclooxygenases (COXs), implicated in the arachidonic acid oxidative metabolism [110]. The lipoxygenase products of AEA are hydroanandamides (HAEAs), which can be formed through the action of 5-, 12- and 15-LOX [110,111]. Unidentified HAEAs have been suggested to bind TRPV1 [112] and PPARα [113]. Moreover, AEA and 2-AG can be enzymatically transformed in prostaglandin ethanolamine (prostamide, PG-EA) and prostaglandin glyceryl ester (PG-G), respectively, through the sequential action of COX-2 and several prostaglandin synthases [114,115].

In the matter of eCBs degradation, FAAH hydrolyses AEA to arachidonic acid and ethanolamine [116]. This enzyme is an intracellular membrane bound-protein belonging to the amidase proteins family [117]. FAAH is expressed in various mammalian tissues, such as brain, testis and liver [116]. Recently, Wey *et al.* [118] have described a second fatty acid amide hydrolase (FAAH2). This gene has been found in humans and multiple primate genomes, but not in some lower placental mammals, including mouse and rat [118]. The first inhibitor proposed for FAAH has been phenylmethylsulfonyl fluoride (PMSF) [119], which is a non-selective serine esterase inhibitor. Other inhibitors have been tested *in vivo*, among which the cyclohexyl carbamic acid 3’-carbamoylbiphenyl-3-yl ester (URB597) displays high selectivity for FAAH [120]. Recently, endogenous molecules, as AEA hydroxyl derivates, able to reversibly inhibit FAAH, have been described [121]. 

However, URB597 administration in the rat brain reduces AEA degradation, but has not effect on 2-AG levels, thus suggesting that 2-AG hydrolysis proceeds through distinct enzymatic pathway [122]. Accordingly, MAGL, a different enzyme responsible of 2-AG degradation, has been isolated [109]. Recently, *magl* has also been cloned and characterised in mouse adipose tissue [109,123] and in rat and human brain [109]. This protein has a cytosolic localisation [109] and has been detected in mouse hippocampus [124]. 

Finally, CBRs, eCBs/CBs, and the machinery for their synthesis and degradation represent a novel signalling system: the cannabinergic system (CS) [125] (for a complete description of CS see Table 3).

**Table 3:** Description of cannabinergic system.

Member	Description	Function	Ref.
**CB_1_**	**Cannabinoid receptor type 1**	**Bind CBs and eCBs**	[15]
**CB_2_**	**Cannabinoid receptor type 2**	**Bind CBs and eCBs**	[16]
**TRPV1**	**Vanilloid receptor**	**Bind AEA**	[61,62]
**EMT**	**Endocannabinoids Membrane Transporter**	**Mediate eCBs diffusion across cellular membrane**	[100]
**NAPE-PLD**	***N-*acylphosphatidylethanolamine** **phospholipase D**	**Biosynthesise AEA**	[86]
**FAAH**	**Fatty Acid Amide Hydrolase**	**Hydrolyse AEA and to a lesser content 2-AG**	[107]
**DAGL**	**Dyacilglycerol lipase**	**Biosynthesise**	[93]
**MAGL**	**Monoacylglycerol lipase**	**Hydrolyse 2-AG**	[108]

Taking in account this background, it is not surprising that endocannabinoid signalling is at the basis of neuroinflammatory diseases (like Alzheimer’s, Parkinson’s and Huntington’s diseases, multiple and amyotrophic lateral sclerosis) [126], cancer cell survival and death [127], immune response [128] and metabolic diseases [129]. For instance, eCBs have been shown to regulate food intake, and in fact SR141716A is an anti-obesity drug for humans. 

Human reproduction is also under the control of endocannabinoid signalling, that regulates the functionality of the hypothalamus-hypophysis-gonads axis and locally the reproductive system with predominant effects on oviductal transport and implantation of embryos - on the female side- as well as spermatogenic output, sperm viability and motility - on the male side. 

In conclusion, eCBs are emerging as widespread signalling molecules, involved to different extents in a plethora of physiological functions in humans.

## 2. Interactions of the Cannabinergic System with Different Neuronal Networks from a Reproductive Perspective

The brain is biologically comparable to a complex architectural structure, found on two main centrepieces: neurons and glia [130]. The first ones control and coordinate body responses to environmental changes communicating each other through the release of excitatory - such as glutamate - and/or inhibitory - γ-aminobutyric acid (GABA) - neurotransmitters. Glia cells perform a number of critical functions, including structural and metabolic support and guidance of development [131].

Substantial lines of evidence indicate that most components of CS are widely expressed in the CNS and their expression pattern reflects the complex repertoire of functions that eCBs perfom in neuronal activity, *via* CB_1_, working as “extracellular retrograde messengers” in GABAergic and glutamatergic synapses. In detail, postsynaptic depolarisation leads to the release of eCBs that in turn activate presynaptic CB_1_ receptor and transiently suppress inhibitory neurotransmitters release [132,133]. By contrast, CB_2_ has a postsynaptic localisation [134].

The multiple physiological functions charged to CS represent a common strategy highly conserved among different classes of vertebrates. In fact, the use of experimental models other than mammals allows the identification of cross-species similarities/differences and provide insight to an integral compilation of all the well-known facets of the system, over the course of evolution. In addition, non-mammalian vertebrates have been recognised to possess morphological features to better study relationships between different neurotransmitter-neuroendocrine-paracrine systems [135,136] and their gonad/brain architecture is simpler than mammals, thus facilitating morpho-functional studies. In this respect, a comprehensive profile of expression for each component of the system has been outlined in the CNS of many species. In mammals, a topographical distribution of CB_1_ [137], CB_2_ [134] and TRPV1 [138] as well as a quantification of eCBs [139] and the analysis of the main enzymes involved in the biosynthesis and degradation of these molecules [139,140] have extensively been discussed in the brain and in the spinal cord [141,142]. The first report on the occurrence of the CS in the amphibian CNS - considered in this review as low vertebrate exemplary - concerns the urodele, *Taricha* *granulosa* where *cb_1_* (italic indicates data at gene level), similarly to mammals, is highly expressed in the brain [143]. In *Xenopus laevis* CNS, cannabinergic neurons are more numerous in forebrain and in spinal cord. In this respect, the expression and the fluctuation of *cb_1_* during the annual reproductive cycle in total brain, encephalic areas and spinal cord of the amphibian anuran, *Rana esculenta* have recently been demonstrated [144]. Accordingly to the expression patterns observed from fish [145,146] to mammals [137], frog *cb1* is mainly produced in the forebrain and midbrain and its involvement in the neuro-endocrine hypothalamic control of adenohypophysis has clearly been shown [147].

In the context of multi-factorial reproductive scenario, CBs have been described as critical signals of the intricate network that control male and female reproduction, at multiple levels: locally, with direct effects on gonads, and centrally, having as target both the hypothalamus and the pituitary [148]. Not less intriguing is the question of how these molecules might influence sexual behavior itself [149]. 

At present, it is well considered the effect of CBs on hormones known to be involved in the regulation of reproductive functions: exposure to Δ^9^-THC inhibits the release of gonadotropin (luteinizing-hormone, LH), prolactin (PRL), and stimulates the release of the stress responsive corticotropin- hormone [150,151].

The presence of CB_1_ on gonadotropes and lactotropes of the anterior pituitary gland [152,153] has led - at the beginning - to hypothesize that the inhibitory action of CBs/eCBs on hormone secretion had as main cellular site the pituitary [154]. In this respect, a complete distribution of CB_1_ - being the main intermediate of both CBs/eCBs actions - has been reported on different pituitary cell types, in many species [151]. In human pituitary, differently from rodents where CB_1_ colocalises with PRL- and LH-secreting cells, CB_1_ has been localised in the corticotrophs and somatotrophs of the anterior lobe, at low levels in PRL-secreting cells, whereas no immunoreactivity has been found in LH-, follicle-stimulating hormone (FSH)-, and thyroid-stimulating hormone (THS)-positive cells. The neural lobe is devoid of CB_1_ [155]. CB_2_ immunoreactivity has been detected in none of the pituitary lobes analysed [33]. Moreover, pituitary *cb_1_* expression is, itself, under the control of sex steroids - androgens and estradiol (E_2_) - in both male and female rodents and male animals have higher levels of *cb_1_* transcripts than females [153]. CB_1_ localisation in the pituitary has widely been described in low vertebrates, as well. In particular, in *X. laevis*, CB_1_ has been found to co-distribute with PRL cells, to be close to LH-secreting cells and absent in the ventro-rostal area of the anterior lobe, where adenocorticotropin hormone (ACTH)-secreting cells are concentrated [156]. Although the hypothalamus contains fewer cannabinoid binding sites compared to other encephalic areas, the activation of CB_1_ in this neuronal district highlights that CBs/eCBs have sites of action upstream of the pituitary. 

Administration of Δ^9^-THC (10 mg/kg weight) and related eCBs/CBs, such as AEA and AM356, inhibits PRL secretion [151,157]. Specifically, the effect of Δ^9^-THC is biphasic, with an early stimulation that precedes the classical inhibitory effect; only this last one is mediated by CB_1_ activation, as demonstrated by its pharmacological blockade with SR141716A [157]. Female rats are unresponsive to Δ^9^-THC administration during proestrus and exhibit an increase in plasma PRL levels during the afternoon of estrus [158]. These ovarian phase-dependent changes in responsiveness to Δ^9^-THC might be due to several sexual dimorphisms of CBs binding sites that fluctuate during the ovarian cycle [159]. Δ^9^-THC administration to ovariectomised (OVX) or hypophysectomised female rats or to dispersed pituitary cells in culture has not effect on PRL release, suggesting that cannabinoid inhibitory effect targets the CNS directly [160]. Dopamine turnover in the tubero-infundibular neurons which express CB_1_, is the suggested neuronal circuitry responsible of such an inhibition [161]. Moreover, PLC activation and Ca^2+^ currents inhibition potentially mediate the action of CB_1_ upon PRL release [162]. In OVX rats, AEA microinjection is not able to significantly modify plasma PRL levels, whereas the same treatment carried out on estrogen-primed OVX rats increases plasma PRL levels, suggesting an effect of AEA modulated by estrogens [161].

The temporary inhibition of LH release is another well determined effect of CB_1_ activation by AEA or Δ^9^-THC [39,150]. In women smoking a single marijuana cigarette with a fixed content of Δ^9^-THC, a decrease of LH has been observed in the luteal phase, whereas no effect has been seen in the follicular phase or in the postmenopausal state [163]. CBs have been shown to decrease LH in male rats, as well [164]. Low dose AEA (0.01 mg/kg) also decreases serum testosterone (T) levels [39], with consequent suppression of spermatogenesis and reduction of testis and accessory reproductive organs weight [165]. A general consensus attributes the inhibitory action of eCBs/CBs on LH release to a suprapituitary site of action: both AEA and ethanol exert, in fact, their pharmacological effects directly upon gonadotropin-releasing hormone 1 (GnRH1) release from the hypothalamus [166]. This inhibition is not completely reversed by AM251, a CB_1_ antagonist, demonstrating that together with the inhibitory CB_1_-dependent pathway, a second inhibitory pathway, probably opioid system-dependent could exist. Anyway, the activation of two neurotransmitters, such as β-endorphin and GABA, has been shown as the essential mechanism for GnRH release suppression. Despite that, the authors have not observed any co-localisation of CB_1_ with GnRH1 neurons [166]. Furthermore, sex steroids, such as estrogens, reverse the inhibitory effect of AEA on GnRH1 secretion [167]. 

Several pieces of evidence indicate the lack of effects by CBs/eCBs on FSH secretion and/or release [168]. Accordingly to the above mentioned evidences, GnRH - the major neuroendocrine initiator of the hormonal cascade controlling reproduction [136] - might represent the central target of eCBs/CBs effects. In detail, elegant studies recently reported by Gammon *et al.* [169] - carried out on immortalised hypothalamic GnRH neurons - document well this regulation pathway. These cells possess a complete and functional CS: that is, they are able to synthesize, degrade and, presumably, transport eCBs across PM. In addition, they contain transcripts for CB_1_ and CB_2_ receptors and the stimulation of these receptors inhibits GnRH secretion. *In vivo* experiments reveal that even if few hypothalamic GnRH neurons contain *cb_1_* transcripts, many neighboring cells possess considerable levels of *cb_1_* transcripts. Moreover, *cb_2_* is expressed in 25% of native hypothalamic GnRH neurons [170]. These data reinforce the idea that eCBs/CBs may perturb reproduction through a direct action - mediated by CBRs activation - upon hypothalamic GnRH neurons or regulating neuronal systems involved in the inhibition (such as GABA) or stimulation (such as glutamate) of GnRH-secreting neurons [150].

In line with this study, key features of a possible crosstalk between GnRH and CB_1_ have been described, using as animal model the green frog *R. esculenta* [147]. Amphibian animal models are very useful since their brain presents a typical laminated structure that is an archetype of those more elaborated of the higher vertebrates [171] and it is characterised by a well defined fluctuation of GnRH production during the annual reproductive cycle [172]. *Cb_1_* and *gnrh1* mRNA expression profiles have been compared in the frog forebrain during the annual sexual cycle revealing a clear mismatch [147,173]. In agreement with these results, a global picture of CB_1_ protein fluctuation in both telencephalon and diencephalon has also been described outlining as general view that GnRH release correlates with minimal levels of CB_1_ [173]. To gain a better knowledge, the morpho-functional relationship between CB_1_ and GnRH1 has been explored in the forebrain. A close contiguity of these two signalling systems has been shown: in particular, the presence of CB_1_ receptor has been ascertained in a subpopulation (20% of total GnRH1 secreting neurons) of the septal and preoptic GnRH1 neurons [147]. Another major outcome of this study is that *in vitro* incubation of male frog diencephalons with AEA (10^-9^ M) clearly reduces *gnrh1* mRNA expression *via cb_1_* activation, as demonstrated by its pharmacological inhibition using SR141716A. Then, a GnRH1 analog (buserelin, 10^-6^ M) inhibits *gnrh1* mRNA synthesis, inducing - in the meantime - *cb_1_* transcription. Therefore, a possible crosstalk - in terms of negative modulation of GnRH neuronal activity by CB_1_ receptor - may be postulated at the basis of gonadotropic pituitary functions, in vertebrates.

Another interesting digression worth of note is how metabolic regulators of the energy balance relay energy status information to the reproductive axis. Successful breeding cycle, gestation and lactation are typical body statuses that need energy. Therefore, GnRH1 neurons need to take into account metabolic status before initiation of reproductive life. Alterations of fertility linked to conditions of disturbed energy balance in humans - from anorexia nervosa to obesity - are, in fact, well-known [174]. Signals coming from various peripheral organs are mainly conveyed at the hypothalamic level to constantly inform the brain about the state of nutrition [175]. 

The central nucleus of this matter is a putative leptin-kisspeptins-GnRH pathway. At the basis of such a peripheral control is located the adipocyte-derived hormone leptin. This 167 amino acid peptide hormone, that reflects the amount of body fat, profoundly affects reproduction exerting its biological effects via interaction with the leptin receptor (Ob-R) [176]. Leptin is a suggested modulator of oocyte quality [177], ovarian function [178], sperm concentration and hormones levels [179]. In the ARC and preoptic area (POA), the conduit for leptin regulation of GnRH/gonadotropin secretion is represented by kisspeptins-secreting neurons, since they express Ob-R [180]. Kisspeptins are a novel family of structurally related peptides encoded by the kiss1 gene, with ability to bind and activate the G protein-coupled receptor, GPR54 [181]. Otherwise, an important role of KiSS-1 has been hypothesised in the metabolic control of fertility, as expression of *kiss-1* gene at the hypothalamus is down-regulated in conditions of negative energy balance and kisspeptin administration is capable of overcoming the hypogonadotropic state observed in undernutrition and disturbed metabolic conditions [182].

The puzzle is complete whether the eCBs/CBs “wedge” is correctly collocated: hypothalamic eCBs appear to be under negative control by leptin, in fact a treatment with leptin - a positive regulator of reproduction - reduces AEA and 2-AG content [183]. Otherwise, obesity is associated with a chronic hypothalamic over-activation of the CS as much as a long period of diet restriction has been associated with reduced levels of 2-AG in the hypothalamus [184].

In the reproductive field, compelling evidences indicate that eCBs/CBs affect sexual behavior in many species. In female rodents, Δ^9^-THC has been reported to facilitate sexual behavior [185]. In detail, a complicate crosstalk between steroid hormones, such as estrogen (E) and progesterone (P), and neurotransmitters, such as dopamine (D), is critical in controlling important neurobehavioral activities [186]. Δ^9^-THC facilitation of sexual receptivity is mediated by CB_1_ and requires a crosstalk between CB_1_-initiated and both P and D-dependent signalling pathways [185]. A positive effect of Δ^9^-THC on female receptivity has also been demonstrated in hamster [187]. Conversely, the treatment of male newts with 5 µg of cannabinoid agonist, levonantradol, significantly reduces spontaneous locomotor activity and courtship clasping behavior [143,188,189]. In *X. laevis*, the use of a rich repertoire of vocalisations in intra-species communication has been clearly ascertained and represents an essential mean to coordinate courtship and male-male dominance behaviors [190]. Neuronal circuitry that is at the basis of calling patterns has also been identified [191]. In particular, the anterior preoptic area (APOA) has been suggested to be a key way station in the activation of calling [192]. More pertinently, APOA has also been indicated as one of the major site of CS localisation [193]. Moreover, important results recently reported by Brahic *et al.* [192] suggest that GnRH neurons could play neuromodulatory roles in vocal centers as well [194]. Furthermore, *X. laevis* hindbrain has been candidated [195] as one of the major encephalic area that generates and coordinates distinct vocal patterns. The idea that GnRH2, functioning as neuromodulator/neurotransmitter, could regulate sexual behavior - a process which also involves CB_1_ [143] - with its strong expression in the posterior areas of the brain [136], is in good agreement with the above mentioned evidences. Anyway, even if *gnrh2/cb_1_* mRNA fluctuations have been delineated in *R. esculenta* mesencephalon/romboencephalon [196], no clear relationship between their expression patterns has emerged to date. 

Stress is known to negatively modulate many aspects of vertebrate physiology and behavior on reproductive functions. Centrally, the stress activates hypothalamic-pituitary-adrenal (HPA) axis, inducing hypothalamic corticotropin-releasing hormone (CRH) production which, in turn, leads to increased circulating levels of ACTH and, finally, of glucocorticoids (GCs) secreted by the adrenal gland [197]. In male mammals, systemic GC administration inhibits circulating gonadotropin levels, decreases seminal vesicles weight [198], and results in fewer implantation sites and viable fetuses in female mates [199]. Anyway, the suppression of hypothalamic-pituitary-gonad (HPG) axis activity by GC is mainly connected to the inhibition of GnRH secretion [200], mediated by a recently-discovered hypothalamic RFamide peptide gonadotropin-inhibitory hormone (GnIH) that inhibits gonadotropin synthesis and secretion [201,202].

In recent years, the CS has emerged as an important regulator of the stress response and a candidate mediator of the stress adaptation [203]. On this basis, AEA has been shown to significantly increase plasma ACTH and corticosterone (CORT) concentrations, even at low dose (0.01 mg/kg), in both wild-type and CB_1_ KO mice. These mice have been generated by Ledent *et al.* [204]. Furthermore, CB_1_ and TRV1 antagonists do not block AEA effects [205]. Moreover, CRH activates two distinct GPCRs, CRH receptor type 1 (CRHR1) and type 2 (CRHR2), strongly expressed in the brain; in particular, CRHR1 has been found at high levels in the hippocampus, cortex and cerebellum and colocalises with CB_1_ in cortical areas [206,207]. It is well known that stress habituation - a term commonly used to explain a decrement in response intensity to a repeated stimulus - involves both a decrease in the activation of HPA axis and a subsequent increase in basal HPA tone [208]. AEA and 2-AG signalling differently contributes to these changes. In particular, repeated stress increases 2-AG content in the amygdala; this increase contributes to the decline in HPA response. Additionally, a reduction in cortolimbic AEA content contributes to the increase in basal HPA tone that accompanies the expression of stress HPA habituation [209]. The influence of CBs signalling on the HPA axis activity is still controversial. In fact, some studies have demonstrated that pharmacological administration of FAAH inhibitors has been proposed as treatment for anxiety-related disorders since their ability to reduce restraint-induced CORT release [210]. Accordingly, CRH-mediated induction of intracellular signalling pathways is inhibited by the activation of the CS [211].

Given its numerous roles in maintaining normal physiological functions and modulating physiopathological responses throughout the CNS, the CS is an important pharmacological target amenable to manipulation directly by CBRs ligands or indirectly by drugs that alter eCBs synthesis and inactivation. In this respect, pharmacological manipulation of AEA and 2-AG signalling, through the main inactivating enzymes, is currently in development and should prove to have significant therapeutic applications in disorders linked to endocannabinoid signalling [212]. One way to alter this signalling is, in fact, to regulate the events responsible for termination of the eCBs uptake and metabolism. Moreover, compounds that selectively manipulate the action and levels of eCBs at their targets have been and are being developed, and represent templates for potential new therapeutic drugs [212].

## 3. The Cannabinergic System in the Male Reproductive Tract: From Spermatogenesis to Sperm Physiology

### 3.1. Testis

In vertebrates the testis contain two discrete morphological compartments: interstitial tissue and seminiferous tubules [213]. The interstitial tissue is primarily composed of vascular, lymphatic and connective tissue elements, macrophages, fibroblasts and the androgen secreting interstitial Leydig cells (LCs) [213]. The seminiferous epithelium contains the differentiating germ cells, supported and protected by Sertoli cells (SCs). It has been reported that THC, in rat isolated SCs, reduces the FSH-induced accumulation of cAMP at concentrations which were neither cytotoxic nor affected cellular ATP levels [214]. This effect can be explained by the activation of CBRs, which, in turn, inhibits adenylyl cyclase, as earlier mentioned [20]. Spermatogenesis requires a continuum of germ cell differentiation, which occurs in three principal phases: the mitotic renewal and proliferation of spermatogonia, meiosis and spermiogenesis [215,216]. In humans chronic exposure to or use of CBs affects quantity of SPZ produced by the testis [217], depresses spermatogenesis [218], decreases T production and secretion by LCs [219], reduces the weight of testes [165] and accessory reproductive organs [220,221]. 

Spermatogenesis is finely regulated by gonadotropins, steroid hormones, paracrine and autocrine factors. Among these factors, eCBs have been described as an emerging class of lipid mediators involved in male fertility [222]. Endocannabinoids are synthesised by gonads. Indeed, AEA has been isolated in rat testis [92], whereas mouse testis contains significant amounts of AEA and 2-AG [223]. Testicular AEA derives from NAPE-PLD activity [86], which shows a higher gene expression in murine isolated SPC and SPT [69]. However, during spermatogenesis, AEA levels remain constant, because also *faah*, the principal AEA degradating enzyme, presents a transcriptional increase during meiosis [69]. Conversely, testicular 2-AG, derived from DAGL α and β (2-AG biosynthetic enzymes), shows high levels in SPG and a dramatic decrease in isolated meiotic SPC and post-meiotic SPT, thus suggesting a role for 2-AG as an autocrine mediator during spermatogenesis [69]. This decline is due to the reduction of the transcriptional levels of the 2-AG biosynthetic enzymes in murine isolated SPC and SPT, and to an increase of 2-AG degradating enzymes (MAGL and FAAH) in the same germ cells [69]. Altogether, these results show that mouse germ cells (SPG, SPC and SPT) have the biochemical tools to produce and inactivate eCBs [69]. With respect to FAAH, this enzyme has been immunolocalised in SPC, elongating SPT (eSPT) and spermatozoa (SPZ) in the testis of the amphibian anuran *R. esculenta* [224]. Accordingly, in rodent testis, FAAH is expressed not only in SPC, SPT, but also in SCs and LCs [223,225].

Isolated murine immature SCs are able to synthesise eCBs [226,227]. Sertoli cells, in fact, show detectable levels of AEA, synthesised *de novo* by NAT and NAPE-PLD activities [227], and degraded by FAAH. Conversely, the endocannabinoid 2-AG, has not been isolated in SCs, but 2-AG metabolic enzymes (DAGL and MAGL) are, instead, active [227]. Moreover, in these cells AEA (but not 2AG) is able to induce DNA fragmentation, thus presenting *in vitro* apoptotic activity in SCs [226]. In order to avoid this pro-apoptotic activity, AEA content is significantly reduced by FSH, through PKA and aromatase-dependent activation of FAAH [227]. Indeed, FAAH is the only target of FSH among the elements of the CS, since FSH enhances FAAH activity and expression, whereas NAT, Nape-PLD, MAGL and DAGL activities are not affected [227]. Therefore, it is reasonable to assume that AEA endogenous tone may control SC population physiologically.

It is worth noting that only immature SCs proliferate in response to FSH, thyroid hormones and various paracrine growth factors, thus determining their final number before adulthood [228]. In turn, SCs number will determine the number of germ cells that can be supported through spermatogenesis and, hence will numerically determine the extent of sperm production, a factor with obvious bearing on fertility [228]. Moreover, it is well known that the neonatal suppression of FSH concentration, in rodent models, significantly reduces the final number of SCs [229]. Therefore, it is likely that the control of SCs number in immature animals by FSH is FAAH mediated.

As mentioned earlier, AEA binds CBRs, which are both expressed in the testis. Indeed, *cb_1_* gene has been cloned and characterised in *R. esculenta*. In detail, *cb_1_* mRNA has been identified in the CNS and in the testis, during the annual reproductive cycle of R. esculenta [144], a seasonal breeder, characterised by a period of resumption of spermatogonial proliferation (late winter to early spring), a well-defined period of mating (March-April), and a postreproductive period [230]. Testicular *cb_1_* profile seems to be well correlated with plasma and intratesticular T levels measured during the year [231,232]. Intriguingly, cDNA obtained from frog brain and testis show nucleotide changes in cDNA sequences compared to genomic DNA [233]. Such differences are not due to multiple polymorphisms, but represent alternative splicing forms of the same gene. This finding is particularly interesting, because different *cb_1_* cDNA sequences, with different mRNA folding and stability, have been identified in frog brain and testis, thus suggesting the possibility of multiple *cb_1_* forms with a tissue specificity, as also reported in mammals. In humans, indeed, *cb_1_* nucleotide changes have been associated to many behavioral/neurological diseases [234,235].

Accordingly, in *R. esculenta* testis, the expression profile of CB_1_ (mRNA and protein) during the annual sexual cycle shows higher levels in September-October period, when seminiferous epithelium presents a massive number of eSPT and newly formed SPZ [144,224,236]. In mouse isolated germ cells, CB_1_ mRNA is expressed in SPG, but gradually increases in purified SPC and SPT, whereas low CB1 mRNA levels have been identified in purified SCs [69]. In the same preparations of purified germ cells, CB_1_ protein show a very faint signal in SPG and SCs, and a gradual increase in meiotic and postmeiotic germ cells extracts [69].

Considering *in toto* murine testes, CB_1_ protein is expressed in SPG, SPC [237], SPT [223] and LCs [39,223]. However, CB_1_ immunolocalisation in SPG is quite controversial, because CB1, a 7-TM receptor, appears in the nuclear compartment. In rat tubular epithelium, CB_1_ is immunolocalised in SCs and in round spermatids (rSPT) until their differentiation in SPZ [225]. It is worth noting that murine immature cells do not express CB_1_ [226]. This discrepancy, apart from species differences, is probably induced by germinal cell contact. Moreover, CB_1_ expression in rat rSPT and eSPT is limited to the acrosomal region [238], thus suggesting the CB_1_ involvement in acrosome and cellular shaping. In rat interstitial compartment, CB_1_ is expressed in LCs [39,223,225], through their differentiation from mesenchymal-like cells to adult LCs (ALC) [225]. In particular, at 41 *days post partum* (dpp), when the unique mitotic division, characterizing the differentiation of immature LCs in ALC, occurs [239], immature mitotic LCs do not immunoexpress CB_1_, suggesting the CB_1_ involvement in the final step of ALC differentiation [225]. To pursue these results further, the LCs count in WT and CB_1_KO mice demonstrates that, in CB_1_KO testes, the number of ALC is significantly lower than in WT mice [225]. Altogether these findings strongly indicate that CB_1_ absence mainly affects ALC proliferation [225]. Accordingly, few ALC may explain the lower *in vitro* basal T secretion in CB_1_KO testes when compared to WT animals [39].

Concerning CB_2_ receptor, a novel testis isoform (*cb_2_A*) with a starting exon located 45 kb upstream from the previously identified promoter transcribing the spleen isoform (*cb_2_B*) was discovered [240]. *Cb_2_A* is highly expressed in testis and brain, whereas *cb_2_B* is more expressed in other peripheral tissues [240]. As for CB_1_, the presence of testis specific isoform is intriguing, because it could help to design drugs directed toward the brain isoform without side effects on testis. 

Mouse isolated germ cells show elevated CB_2_ transcriptional levels in all stages of spermatogenesis (SPG, SPC and SPT) with a relative peak of expression in SPC, whereas a purified preparation of SCs present low *cb_2_* mRNA levels [69]. The presence of CB_2_ protein has also been confirmed by immunofluorescence. Indeed, a strong signal has been detected in differentiating SPG and SPC, whereas a weak signal appears in SPT [69]. Additionally, in mouse isolated SPG, CB_2_ activation, through a specific agonist, exerts a pro-differentiative effect. As mentioned earlier, 2-AG is the mostly abundant endocannabinoid in SPG, therefore it is possible to speculate that it promotes, through CB_2_, the spermatogonial progression toward meiosis [69]). Also murine isolated immature SCs express a functional CB_2_ receptor, as suggested by binding assays [226]. However, the specific proapoptotic effect of AEA is not mediated by CB_1_, CB_2_ or TRPV1 receptors [226]. Accordingly in mouse testis, CB_2_ has been immunolocalised in SPC and in SCs encircling SPC and SPT, whereas LCs are negative [223]. 

Regarding the TRPV1 channels, a strong increase of mRNA expression has been observed in SPC and SPT, in agreement with an increase of TRPV1 protein from meiotic germ cells to differentiating rSPT [69]. A recent report shows that TRPV1 protects germ cells against heat stress and plays a protective role in meiotic progression [241]. Moreover, murine isolated SCs express low mRNA and protein TRPV1 levels [69]. However, this channel is functional, as demonstrated by binding activity assay [227]. 

Taking in account the above described background, it is possible to explain many effects of THC on male reproduction. However, it is still unclear whether the reported effects of *Cannabis* on male sexual and reproductive function may result from direct inhibition of testicular spermatogenesis and/or steroidogenesis, through their cognate receptors, or whether some effects may be due to altered hormone levels, which are necessary for supporting male reproduction. Nevertheless, it should be considered that many of the effects on the endocrine system caused by chronic treatment with THC are completely reversible with time, suggesting that tolerance develops with acute exposure to THC [217].To solve this question, a CBR tissue specific KO mouse may be useful to address if the cannabinoid actions on testis are direct or indirect. 

### 3.2. Excurrent duct system

Once formed within the seminiferous tubules, the immotile SPZ are released into luminal fluid (spermiation) and transported to the excurrent duct system, differentially organised according to the species [196]. In mammals, sperm are released in the epididymis, where they undergo many maturational changes to attain the capacity to fertilise the oocyte [242]. Sperm maturation is not intrinsic to sperm themselves but it is acquired during their transit through the epididymis [243].

The epididymis is a long convoluted tube with three main regions, named *caput*, *corpus* and *cauda*, where SPZ undergo numerous membrane modifications (collectively known as capacitation), before they interact correctly with the oocyte within the female reproductive tract [244]. Capacitation comprises a series of processes, such as modifications in sperm surface protein distribution, alterations in PM characteristics, changes in enzymatic activities and modulation of intracellular constituents [242]. In mammals, the motility waveform changes when SPZ enter in the female reproductive tract, with increases in both the amplitude and asymmetry of flagellar bending. These changes result in a whiplash-like motion, termed hyperactivated motility, which facilitates sperm transport in the oviduct [245]. Herein, SPZ undergo the acrosome reaction (AR), which results in the activation and release of acrosomal enzymes, thus allowing SPZ to bind and penetrate the zona pellucida (ZP), and to fuse with the oocyte PM [246]. 

Recent findings have demonstrated that the murine [223,247], boar [218] and human reproductive tracts [248] contain eCBs, suggesting the pivotal role of these lipid mediators in multiple physiological processes of male reproductive system. 

**Figure 1:**
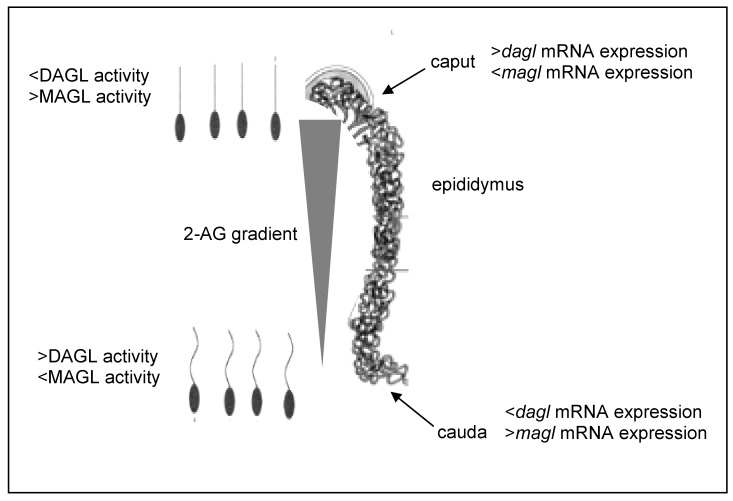
Treadmilling activity of MAGL in the cauda epididymis: high *magl* expression in the epididymis drives the 2-AG passage from actively biosynthesising *cauda* SPZ to epididymal epithelial cells, determining the 2-AG gradient.

In mouse epididymis, the levels of 2-AG, but not AEA, dramatically decrease from *caput* to *cauda* [247]. Moreover, the *dagl* mRNA expression decreases in *cauda* epididymis, whereas *magl* mRNA expression increases in the same epididymal segment [247]. By contrast, the DAGL enzymatic activity significantly increases in *cauda* SPZ, whereas MAGL activity decreases [247]. Altogether these results suggest that the 2-AG gradient is probably due to a “stripping” of 2-AG from SPZ mediated by epididymis. Specifically, the high expression of *magl* in the epididymis could be responsible of 2-AG passage from 2-AG actively biosynthesising *cauda* SPZ to epididymal epithelial cells [247] (Figure 1). Accordingly, *in vivo* treatments with AM404 and OMDM-1, two inhibitors of endocannabinoid cellular uptake, significantly increase 2-AG content in *cauda* SPZ, thus reducing SPZ motility [247]. As consequence, these results strongly suggest that the 2-AG gradient, along epididymis, induces caudal SPZ to acquire potential motility (“start up”), in fact any alteration in 2-AG content in the epididymal milieu, affects sperm motility [247]. Conversely, mice with genetic loss of FAAH present high epididymal AEA levels in comparison to WT animals [223]. However, also high AEA content in the epididymis induces a sluggish motility in FAAH null sperm, when incubated in capacitated medium [223], thus suggesting that any alteration of the eCBs tone during the epididymal transit negatively affects sperm motility. In this respect, it is reasonable to hypothesize that, also in humans, alterations in the eCBs gradient along epididymis may explain some cases of male idiopathic infertility, where an impairment of sperm motility is observed. Consequently, a screening of eCBs tone in these patients may be useful to determine the correct pharmacological approach, and may be introduced in the common parameters evaluated in semen analysis. Moreover, the increasing percentage of male idiopathic infertility may be also explained by the more diffuse recreational use of *Cannabis*. Indeed, male rats after a single THC administration, present higher THC concentration inside epididymal fat than in brain or testis, where blood brain and testis barriers work efficiently [249]. THC, in fact, is easily stored in fat tissue, thanks to its lipophilic nature. Therefore, in men exposed to marijuana, THC may accumulate in epididymal fat tissue and damage sperm maturation. 

A complete CS related to AEA has been also characterised in boar [68] and human SPZ [250]. Indeed, human SPZ, as boar SPZ, express the CS enzymes involved in AEA synthesis (NAPE-PLD) and hydrolysis (EMT and FAAH) [68,250]. The immunofluorescent analysis localises NAPE-PLD and FAAH on the post acrosomal region of the sperm head and on the whole middle region in boar [68] and human SPZ [250]. Additionally, in boar SPZ, both NAPE-PLD and FAAH are active enzymes, which regulate the endogenous AEA tone in these cells [68]. 

Nevertheless, eCBs need CB_1_ and CB_2_ receptors to regulate sperm maturation during epididymal transit. Both receptors are expressed both in SPZ and epididymal epithelial cells [223]. CB_1_ has been evidenced in mammalian [68,223,225] and, in particular, human SPZ [251,252,253]. In detail, immunofluorescent analysis demonstrates that CB_1_ is present in the head, close to the acrosome, and midpiece of human [250,253], boar [68], mouse [223] and rat SPZ [225]. It is noteworthy that an ultrastructural analysis on human SPZ, through transmission electron microscopy, immunolocalises CB_1_ on the membranes of head and on the mitochondria in midpiece [252]. Recently, a functional CB_2_ receptor has been also detected in human SPZ [254], where it is localised in the postacrosomal region and tail of sperm cells [254].

Additionally, boar and human SPZ express TRPV1 channel, which is localised in the postacrosomal region of sperm head [68,250]. According to CB_1_, CB_2_ and TRPV1 localisation in the acrosomal region, midpiece and flagellum of mammalian sperm, it has been suggested that eCBs, through their cognate receptors, may influence sperm motility [225,247,253] and acrosome reaction [68,255,256].

Although quiescent in the epididymis, mammalian sperm display vigorous flagellar movement, immediately upon collection into physiological medium [245].

Experiments with CB_1_KO mice [257] and mice treated with AM281, a CB_1_ antagonist, [222,247] show that CB_1_ lack or inactivation clearly increases the percentage of motile SPZ in *caput*, which becomes comparable to that observed in the *cauda*, suggesting that CB_1_ signalling controls the number of motile SPZ along the epididymus by keeping quiescent sperm motility in the *caput* [238,247,257]. As reported earlier, the 2-AG (but not AEA) levels dramatically decrease from *caput* to *cauda* SPZ, supporting the hypothesis that the increased percentage of motile SPZ collected from the *cauda* is caused by decreased levels of 2-AG, and, in turn, by a reduced CB_1_ activity [247]. Indeed, *in vitro* studies show that AEA inhibits, in the same way, the motility of human [253] and frog ejaculated SPZ [224] and mouse epididymal SPZ [247] through CB_1_ receptor, without any toxic effect on these cells. At concentrations up to 1 μM, in fact, AEA signalling inhibits sperm motility, leaving sperm viability unaltered [247,253]. Conversely, a recent report shows that Met-F-AEA, a metabolically stable analogue of AEA with greater receptor affinity for CBRs, induces SPZ death at lower concentration (0.1 μM), compromising their fertilizing capacity [258]. This effect may be due to greater receptor affinity for CBRs than other congeners. 

Interestingly, while CB_1_ selective agonists increase the number of immobile sperm cells, *in vitro* incubation of human ejaculated SPZ with selective CB_2_ agonists significantly increases the slow/sluggish progressive sperm cell population, thus suggesting that CB_2_ regulates human sperm motility in a distinct manner in comparison to CB_1_ [254]. The different localisation and function of CB_1_ and CB_2_ in SPZ is also correlated to the compartimentalisation of ATP sources within the flagellum of the SPZ. Indeed, glycolysis is restricted to the principal piece, which is the longest segment of sperm flagellum. In contrast, oxidative phosphorylation is confined to the proximal segment of the flagellum, where mitochondria are located (middle piece) [259].

It has been demonstrated that most of the energy required for sperm motility is generated by glycolysis [260]. In detail, because the flagellar motion of sperm lacking glyceraldehyde 3-phosphate dehydrogenase S (GAPDS), a sperm specific glycolytic enzyme, is quite sluggish and rarely results in forward movement [260], as previously reported in human SPZ treated with CB_2_ selective agonists, we can hypothesize that these agonists influence glycolytic pathway. Conversely, the inhibitory effect on sperm motility with CB_1_ agonists could address for a combined action on glycolysis and oxidative phosphorylation. Recently, in human ejaculated SPZ, AEA, through CB_1_, has been reported to inhibit mitochondrial activity in a dose dependent manner [253]. In detail, AEA treatment decreases, in a rapid way, mitochondrial rhodamine (R-123) uptake, which has been established to be highly sensitive to factors directly reducing the mitochondrial membrane potential of sperm [261].

Additionally, in uncapacitated human sperm, Met-F-AEA has been reported to reduce the percentage of motile SPZ, independently by CBRs. Indeed, Met-F-AEA treatment, by activating the glycogen synthase [262] through glycogen synthase kinase 3 (GSK-3) phosphorylation [252], induces the accumulation of glycogen and makes glucose unavailable for glycolysis.

Furthermore, THC concentrations up to 0.8 μM have been detected in peripheral blood of subjects after marijuana smoking [263]. These plasma concentrations of THC resemble the AEA concentration (1 μM) used to alter sperm motility and mitochondrial activity. Accordingly, human sperm, incubated with THC at concentrations equivalent to therapeutic and recreational plasma levels, showed a significantly decrease in progressive motility and straight line velocity [264]. Moreover, CBs (Δ^8^-THC and Δ^9^-THC) are potent inhibitors of mitochondrial oxygen consumption in human washed spermatozoa, probably through a direct effect on mitochondrial respiratory chain. Conversely, in neat semen, mitochondrial respiration is less affected by CBs treatment, thus suggesting the presence of protective factors in seminal plasma [265].

In mouse SPZ, TRPV1 activation by SR141716A and CPS, decreases the percentage of motile SPZ only in *cauda* epididymis, suggesting that both CB_1_ and TRPV1 receptors might induce SPZ to acquire potential progressive motility in the *cauda* epididymis [247]. Therefore, CB_1_, CB_2_, and TRPV1 are important in mediating SPZ functions. Recent data also suggest that CB_1_ is also involved in downstream events of sperm physiology. Specifically, after mating, mammalian SPZ are stored in the isthmic region of the oviduct through adhesion to the epithelial cells under conditions that maintain sperm viability and fertilisation competence until ovulation takes places [242]. Current results indicate that AEA is involved in bovine sperm-oviduct interaction. Indeed, methanandamide (Met-AEA), a non-hydrolysable AEA analog, inhibits, through CB_1_ receptor, sperm binding to and induces sperm release from oviductal epithelia. This effect is not caused by inhibition of sperm progressive motility or by induction of AR, suggesting that AEA modulates the sperm-oviduct interaction [266].

As the sperm approaches the ZP of the oocyte, the membrane surrounding the acrosome fuses with sperm PM, exposing the acrosome proteolytic enzymes necessary for penetration of the oocyte coats [242]. Boar SPZ, incubated under capacitating conditions in the presence of Met-AEA, fail to undergo ZP induced AR. This inhibitory effect of Met-AEA depends on its ability to reduce intracellular levels of cAMP, a typical CB_1_ mediated effect [267]. Also, in human sperm, AEA inhibits ZP-induced acrosome reaction, even if CB_1_ activation does not induce any variation in sperm intracellular calcium concentrations [253], which is the most important physiological AR regulator [268]. Accordingly, high AEA levels in FAAH null mice reduce the capacity of sperm to penetrate the ZP barrier, probably because the protease activity in the acrosome is unadeguate for penetration in the oocyte or FAAH null sperm do not acquire hypermotility after capacitation [223]. 

In human SPZ, as in boar, TRPV1 activation seems to play a role in preventing spontaneous acrosome exocytosis during capacitation, in fact the specific TRPV1 antagonist, capsazepine (CPZ), significantly increases the incidence of spontaneous AR [68,250]. Moreover, the sperm exposure to OMDM-1, a specific inhibitor of EMT, prevents the promoting effect of CPZ on spontaneous AR rate, by increasing the intracellular AEA content, which, in turn, displaces CPZ from TRPV1 [269,250]. As a consequence, AEA, which increases during sperm capacitation [269], is able to prevent premature AR, thereby promoting sperm fertilizing ability. At this point, it is intriguingly to note that CB_1_ receptor is involved in the control of ZP induced AR, whereas TRPV1 activation regulates the spontaneous AR.

In conclusion, all these findings indicate that CS influences male reproduction at different levels, from spermatogenesis and/or steroidogenesis to sperm maturation. Consequently, all these effects should be carefully weighed against the potential therapeuthical effects in the treatment of obesity and neurological disorders. 

## 4. Effects of the Cannabinergic System on Female Reproduction: From Ovary to Utero-placental Relationship

The effects of *Cannabis* and THC on the human ovary consist in suppression of ovulation [270]. Alteration on E and P production by human placenta has also been reported [271]. During the ovarian cycle plasma LH, FSH and PRL levels are high in the early follicular phase and consequently decrease in the late follicular phase until luteal phase. In particular, the concentration of FSH and PRL shows a similar but less marked change to that of LH throughout the menstrual cycle with a significant decline in the luteal phase of the cycle [272]. Acute administration of THC suppresses LH secretion. In detail, marijuana use during the luteal phase of the menstrual cycle reduces of 30% LH plasma levels, which remain unchanged during follicular phase [163]. Other studies show increased anovulatory cycles and short luteal phases in chronic women smokers [273]. Nevertheless, direct adverse effects on the ovary have clearly been observed as *Cannabis* users present a higher risk of primary infertility due to anovulation [274]. Interestingly, even when these women have *in vitro* fertilisation (IVF) treatment, they produce poor quality oocytes and lower pregnancy rates compared to non-users [275]. 

Additionally, in laboratory animals, THC inhibits the PRL secretion [276] and suppresses the episodic LH secretion [277]. *In vitro* studies in rat ovary demonstrate that THC, when administered on the day of proestrus, exerts a direct inhibitory effect on folliculogenesis [278] and ovulation by suppressing plasma FSH and the pre-ovulatory LH surge, [279,280]. Anovulation has also been observed in rabbits and rhesus monkeys [281] as a result of LH surge disruption [277]. It has been suggested that this may be primarily due to the hypothalamic inhibition of GnRH release [282]. Furthermore, THC has also been shown to cause a dose-dependent inhibition of the FSH-stimulated accumulation of P and E in ovarian granulosa cells [283]. Other studies indicate that embryotoxicity and specific teratological malformations in rats, hamsters and rabbits has been correlated with exposure to natural *Cannabis* extracts during pregnancy [284,285].

Uterus synthesises AEA and the embryos express CBRs; these observations suggest a role for eCBs during early pregnancy [221]. In fact, the CS members have been localised in human ovary; CB_1_ and CB_2_ have been localised in the medulla and cortex and here, in particular, in the granulosa cells of primordial, primary, secondary and tertiary follicles and in the theca cells of secondary and tertiary follicles [286]. Analysis of oocytes at all stages of development shows that oocytes of tertiary follicles express CB_2_, suggesting that they respond to AEA through CB_2_ activation only in the last stage of its development [286]. In this respect, the AEA presence has been demonstrated in ovarian follicular fluid and mid-cycle oviductal fluid, suggesting that the factors involved in the folliculogenesis may also modulate AEA levels in the ovary [248]. Probably, this endocannabinoid might be produced by granulosa cells in ovarian follicles as well as in granulosa cells adjacent at ovulated oocytes, but the mechanisms controlling its production and release are still unknown [248]. Recently, the relationship between AEA, sex steroids and gonadotrophins, during menstrual cycle, has been investigated. AEA peak plasma occurs at ovulation and positively correlates with estradiol and gonadotropin levels suggesting that these may be involved in the regulation of AEA levels [287]. 

However, NAPE-PLD has been found in granulosa and theca cells, while FAAH only in theca cells of secondary and tertiary follicles. Therefore, it is conceivable that the granulosa cells of secondary and tertiary follicles, but not oocytes, produces AEA and that in granulosa cells AEA degradation proceeds following different pathways [286]. These findings indicate that eCBs are involved in oocyte maturation and ovulation.

The following stages in the reproductive events are: fertilisation and formation of blastocyst composed of a hollow sphere of trophoblast cells, inside of which there is a small cluster of cells, the inner cell mass (ICM). Trophoblasts go on to contribute to fetal membrane systems, while ICM is destined largely to become embryo. Between fertilisation and blastocyst formation, the embryo moves out of the oviduct, into the lumen of the uterus [288]. 

In mouse model, embryos, at the late morula or early blastocyst stage, enter in the uterus where develop and differentiate to acquire implantation competence and implant into the receptive uterus [289]. Mouse embryos express both CB_1_ and CB_2_ receptors [290], but also FAAH and NAPE-PLD [221]. In detail, *cb_1_* mRNA has been detected from the late two-cell stage, whereas *cb2* is present from the one-cell through the blastocyst stages. NAPE-PLD protein has been found from the stage of the fertilised egg through to the blastocyst stage, while FAAH first appears in 2-cell embryos, decreases in the morula and its expression becomes more abundant in trophectoderm of blastocysts. The expression of both enzymes is in agreement with their mRNA expression [221]. In particular, increasing FAAH expression in blastocysts suggests that its hydrolytic activity may represent a protective mechanism against an excessive AEA production in these tissues. In fact, given the CS members presence in the blastocyst, mouse embryo represents a target for CBs and eCBs. In this respect, it has been shown that high doses of AEA and 2-AG, *in vitro*, arrest embryo development; this effect has been reversed by CB_1_ antagonists: SR141716A or AM251, but not by CB_2_ antagonist, SR144528, indicating that cannabinoid effects on embryo development are CB_1_ mediated [291]. Moreover, the *in vivo* effects of THC, CBD or CBN on preimplantation embryo development and implantation, in mice, have also been reported [221]. On day 4, mice treated with THC show oviductal retention of embryos with asynchronous development and fail implantation in the uterus. The examination of implantation on day 5, in mice receiving THC in days 1-4, confirms a failed implantation [221]. The CB_1_ involvement in normal embryo growth has also been shown, in fact in CB_1_KO embryos the development becomes asynchronous [292], while CB_1_ heterozygous embryos show normal development [293]. 

Moreover, the CS has also been involved in embryo transport; studies carried out in CB_1_- and CB_2_-KO mice, the latter generated by Buckley *et al.* [294] certainly show that oviductal transport is a CB_1_-dependent mechanism [292,293] and that genetic or pharmacological loss of CB_1_ determines embryos retention in the oviduct [221]. Here, enzymes responsible of AEA synthesis and degradation are present on days 1-4 of pregnancy with an inverse distribution in comparison to embryos. In fact, NAPE expression is higher in isthmus epithelium than in ampulla; whereas FAAH presents inverse expression levels [221]. In the ampulla, high FAAH and low NAPE-PLD determine a low concentration of AEA; on the contrary, in the isthmus low FAAH and high NAPE-PLD maintain high levels of AEA. As a consequence, in mouse embryos and oviducts, a balance between AEA synthesis and degradation is generated by NAPE-PLD and FAAH, respectively. This produces locally an appropriate “AEA tone” for normal embryo development and oviductal transport until the uterus. Pharmacological or genetic suppression of FAAH activity in mouse embryos and oviducts enhances AEA levels *in loco*, thus inhibiting embryonic development, causing embryo retention, impairing implantation and fertility [221]. Besides, it is well-known that embryo transport occurs through a wave of oviduct smooth muscle movement controlled by the sympathetic nervous system [295]. In this respect, it has been observed, in the oviduct muscle, a co-localisation of CB_1_ and α1and β2-adrenergic receptors. This may indicate that the cannabinoid and adrenergic systems coordinate together oviductal motility for normal journey of embryos into the uterus, determining an alternation of contraction ad relaxation of oviduct *muscolaris*. Thus, high AEA levels, through CB_1_, reduces norephinephrine (NE) release from nerve terminals, determining a relaxation of smooth muscle; on the contrary, low AEA levels, enhancing NE release, produce *muscolaris* contraction [293]. Collectively, these observations, in murine model, provide evidence that, while embryonic CB_1_ primarily contributes to normal embryo development, oviductal CB_1_ directs the timely transport of embryos. 

Recently, it has been shown, in human, that aberrant endocannabinoid signalling in Fallopian tube leads to ectopic pregnancy; in fact, *cb_1_* mRNA has been detected at low levels in Fallopian tube and endometrium of women with ectopic pregnancy, if compared to intra-uterine pregnancies. Moreover, a possible association between polymorphism genotypes of *cb_1_* gene and ectopic pregnancy has been investigated [296]. 

Synchronised embryo development to the blastocyst stage, preparation of the uterus to the receptive stage and normal oviductal embryo transport are essential prerequisites for initiation of implantation in uterus [289,297]. Implantation is a process that involves complex interactions between the blastocyst and the uterus; in particular, the embryo establishes a physical and physiological contact with the maternal endometrium, followed by stromal cell decidualisation at the sites of blastocysts [298]. The uterine environment is divided into pre-receptive, receptive, and non-receptive state [297,299]; these three phases of the uterus during pregnancy or pseudopregnancy are sequentially programmed by ovarian P and E [300], which are the primary regulators of uterine receptivity for implantation [301]. In order for implantation to take place, it is very important that the uterus differentiates into the “receptive state” [297]. In fact, there is only a specific period of time during which implantation is possible; this period is defined “implantation window” and represents the time tightly limited, in which the uterus is receptive to accept the blastocyst. In mouse, the uterus in pre-receptive phase becomes receptive in the day of implantation (day 4), when occurs ovarian estrogens secretion, and, by day 5, it becomes non-receptive for blastocyst implantation. Other factors, such as cytokines, growth factors, transcription factors and lipid signalling molecules, participate in these processes, exercising autocrine, paracrine, and/or juxtacrine control [297,302].

Some studies have found that mouse uterine luminal and glandular epithelial cells express *faah* mRNA [303]. Furthermore, FAAH protein expression and activity has recently been localised in endometrial epithelium regions [304]. Therefore, it has been proposed that AEA plays an important role in the local regulation of uterine implantation [292]. In fact AEA levels have been measured in both receptive and non-receptive uteri and they have been demonstrated to be inversely correlated to uterine receptivity for implantation [221]. Lower AEA levels characterize uterine receptive phase in comparison to non-receptive uterus that have higher AEA levels [305]. To understand the mechanisms regulating uterine AEA levels, the expression profiles of *nape-pld* and *faah* have been examined in the uterus. Higher levels of *nape-pld* mRNA and NAPE-PLD activity have been found in non-receptive uteri and in inter-implantation sites, whereas both mRNA and protein levels were lower in implantation sites and receptive uteri [306,307]. These data are in agreement with regulated AEA levels characterizing these tissues. It is interesting that FAAH expression and activity show an inverse relationship, since higher FAAH expression and activity have been observed at implantation sites and in the receptive uteri. Recent evidence suggests that E and P, alone or in combination, down-regulate the expression of NAPE-PLD, through their nuclear receptors [306] and inhibit FAAH activity [304] in mouse uterus. AEA-metabolizing enzymes are regulated by these two hormones also in rat uterus [308]. Since AEA levels depend also on its degradation by COX-2, localisation of this enzyme in the inter-implantation and implantation sites has recently been investigated. COX-2 has been localised in uterus and in the luminal epithelium on day 1 of pregnancy, whereas it is weakly visible in the peri-implantation area [307]. 

AEA, at low concentration, confers blastocyst competency to implantation *via* CB_1_, differentially modulating ERK signalling and Ca^2+^ channel activity. In particular, AEA at a low concentration (7 nM) induces ERK phosphorylation and nuclear translocation in trophectoderm cells, thus allowing blastocyst implantation in the receptive uterus. Conversely, AEA at a higher concentration (28 nM) inhibits Ca^2+^ channels, thus compromising Ca^2+^ mobilisation needed for implantation [309]. These results suggest that low AEA and CB_1_ levels are beneficial to implantation and that low FAAH activity and subsequent increased AEA levels may be one of the causes of implantation failure or pregnancy loss.

In human, it has also been proposed that low plasma AEA levels are required for successful pregnancy progression. In fact, recent observations suggest that in a viable pregnancy, AEA levels fluctuate from the time of ovulation to early pregnancy, with the highest levels at the time of ovulation and the lowest at 6 weeks gestation. Thus, AEA plasma changes become very important to monitor the appropriate timing of embryo transfer in women undergoing IVF/ICSI [310].

During early pregnancy, plasma AEA levels are inversely associated to FAAH activity in maternal lymphocytes, thus suggesting that high AEA levels and low FAAH activity may cause early pregnancy loss and failure to achieve an ongoing pregnancy after IVF and embryo transfer [311]. These blood cells have a critical role in embryo implantation and maintenance of the fetus in humans [312], because they produce leukaemia inhibitory factor (LIF) and immunomodulatory proteins, such as T-helper (Th) 2-type cytokines (interleukin, IL-3, IL-4 and IL-10), which favour foetal implantation and survival [313,314]. In this respect, FAAH in lymphocyte is stimulated by P and Th2 [315]. Th1 cytokines (IL-2, IL-12 and interferon-γ, IFN-γ), as Th2, are released by T-lymphocytes and have different effects on trophoblast growth, because, while Th2 favour implantation by stimulating trophoblast growth, through natural killer (NK) cell activity inhibition, Th1 by activating NK cells, cause a trophoblast damage disadvantaging gestation [312]. Moreover, *in vitro* treatment with AEA of human lymphocytes inhibits LIF release [315]. Additionally, P activates *faah* in human T lymphocytes by enhancing its promoter and thus up-regulating *faah* gene expression [316]. *Faah* activation by P is further enhanced by IL-4 and IL-10, whereas IL-12 or IFN-γ inhibit the AEA-hydrolysing activity. 

Plasma AEA levels decrease from the first through second and third trimester of pregnancy [317]. These levels increase before the onset of clinically apparent labor and during labor, suggesting a role for AEA on the uterus in normal labor [317,318]. Subsequently, it has been investigated whether plasma AEA levels may predict outcome in women presenting threatened miscarriage. These results show that all women who miscarried have AEA values greater than 2.0 nM in comparison to women who have live births [319]. Recently Marczylo *et al.* [320], developed a reliable and reproducible method of solid-phase extraction of AEA and measurement in reproductive tissues to determine AEA concentrations at human maternal:fetal interface at term. AEA levels, both in human placenta, both in fetal membranes, are in the picomole per gram range which is significantly lower than previously observed in other animal and human genital tissues.

Additionally, also *faah* mRNA levels appear to be regulated during gestation: they increase from week 9 week, peaking between weeks 10 and 11 of gestation before declining again by week 12. These findings suggest that placenta may form a barrier to prevent AEA transfer from maternal blood to the fetus and that AEA local levels are modulated by regulation of FAAH expression during gestation [321]. In this respect, it has recently been show that FAAH is absent in trophoblast layers of placental villi from first trimester spontaneous miscarriage, whereas it is present in syncytiotrophoblast and overall in cytotrophoblast of normal placental tissues of matched gestational age [322]. On the contrary CB_1_ expression is higher in placental villi from first trimester spontaneous miscarriage [322]. These data are also in agreement with a previous report [321] and suggest a role for FAAH to prevent detrimental effects of maternal AEA on fetus. In fact, low FAAH and high CB_1_ levels may contribute to spontaneous miscarriage [322]. The placenta expression of *nape-pld* mRNA, has also been shown. In particular, this transcript is present at low levels in spontaneous miscarriage first trimester placenta. Thus, as in embryo development, a critical balance between *nape-pld* and FAAH may create a local AEA tone, essential for fetus protection [322].

CB_1_ and FAAH have been localised also in human term placental tissue: CB_1_ is present with the highest expression in amnion and trophoblast; whereas FAAH is still present in amnion and the decidual layer, but has not been detected in the trophoblast [323]. To better understand eCBs role in the events driving the labouring delivery, Acone *et al.* [324] compare the expression and localisation of CB_1_ and FAAH in placental villous samples obtained from women undergoing elective caesarean section and women having a normal spontaneous delivery, thus characterizing the non labouring-labouring transition. Whereas FAAH is absent in all samples analysed, CB_1_ has been localised in placental villous of both groups, with a higher expression in non-labouring women. This different expression may be useful to explain AEA effects in placental regions during non labouring-labouring transition. Thus, in non-labouring women, placental CB_1_ up-regulation may produce myometrial relaxant factors, such as nitric oxide (NO) and gonadotropin releasing factor, to maintain quiescent the uterus , while, close to the term, in labouring patients the CB_1_ down regulation may reduce these factors production [324]. NO represents an important modulator of cellular responses in many tissues and possesses a vasodilator effect [325,326] to maintain low vascular resistance in the fetoplacental circulation [327,328]. Furthermore, AEA modulates NO synthesis by NOS in rat placenta [329]. Here, AEA, on the one hand, diminishes NOS activity *via* CBR, on the other hand, as an endovanilloid, stimulates NOS activity *via* TRPV1. This dual effect is very important because high levels of NO exert toxic effects, while low levels cause a reduction of the placental perfusion with consequent foetal nutrition decrease.

Finally, a critical event in late human pregnancy, that regulates progression of term and pretermlabour and rupture of membranes, is prostaglandin E_2_ (PGE_2_) production by fetal membranes. It has been showed that eCBs, *via* CB_1_, stimulate PGE_2_ synthesis, through COX-2 induction [330]. CB_1_ also regulates labour by interacting with CRH and CORT endocrine axis. In fact, CB_1_ loss induces preterm birth in mice, influencing CRH and CORT levels during the end of gestation [331]. 

Altogether, these data draw attention to endocannabinoid signalling in different female reproductive events. In particular, new genetic and molecular evidences about eCBs implication in physiology of pregnancy have been provided. Thus, further studies will be useful to explain the role of these emerging molecules in the regulation of fertility and to open new avenues in their pharmacological employment.

## 5. Closing Remarks

During the last decades, a remarkable increase in our understanding of the impact of the cannabinergic system on many physiological functions in vertebrates has been emphasised. Concerning reproduction, the cannabinoids role in fertilisation, preimplantation embryo and spermatogenesis opens emerging prospectives in clinical applications.

In this review, we have analysed the pharmacological basis of this system, by focusing on its involvement in central and peripheral control of male and female fertility, especially in mammals with few hints to amphibian anuran *R. esculenta*, as a simple model of lower vertebrates.

Many pharmaceutical companies have developed more potent synthetic cannabinoid analogues and antagonists to improve infertility and reproductive health. Accordingly, the existence of tissue specific nucleotide changes of CB_1_, observed in *R. esculenta* as in humans, may have an important impact on clinical practice. In this respect, pharmacological production of tissue specific drugs, which target the main components of CS, may represent a new promising therapeutical approach, by allowing a selective action at peripheral organs without side effects in neural circuits that regulate mood and anxiety. 

## Abbreviations

2-AG: 2-arachydonoyl glycerol
2-AGE: 2-arachidonoylglyceryl ether
AC: adenylyl cyclase
ACTH: adenocorticotropin hormone
AEA: anandamide
AM404: *N*-(4-hydroxyphenyl)-arachidonylamide
APOA: anterior preoptic area
AR: acrosome reaction
ARC: arcuate nucleus CB_1_, cannabinoid receptor type-1
CB_2_: cannabinoid receptor type-2
CBC: cannabichromene
CBD: cannabidiol
CBG: cannabigerol
CBL: cannabicyclol
CBN: cannabinol
CBRs: cannabinoid receptors
CBs: cannabinoids
cNOS: nitric oxide synthase
CNS: central nervous system
CORT: corticosterone
COXs: cyclooxygenases
CPS: capsaicin
CPZ: capsazepine
CRH: corticotropin-releasing hormone
CS: cannabinergic system
D: dopamine
1,2-DAG: 1,2-diacylglycerol
DAGL: diacylglycerol lipase
dpp: days post partum
E_2_: estradiol
eCBs: endocannabinoids
EMT: endocannabinoid membrane transporter
ERK: extracellular signal-regulated protein kinases
eSPT: elongating SPT
FAAH: fatty acid amide hydrolase
FABP: fatty acid binding proteins
FSH: follicle-stimulating hormone
GABA: γ-aminobutyric acid
GAPDS: glyceraldehyde 3-phosphate dehydrogenase S
GCs: glucocorticoids
GnIH: gonadotropin-inhibitory hormone
GnRH1: gonadotropin-releasing hormone 1
GPCR: GTP-binding protein-coupled receptors
GPR54: G protein-coupled receptor 54
GPR55: G protein-coupled receptor 55
GPR119: G protein-coupled receptor 119
GSK-3: glycogen synthase kinase 3
HAEAs: hydroanandamides
HPA: hypothalamic-pituitary-adrenal
HPG: hypothalamic-pituitary-gonad
HU-210: 11- hydroxy-Δ^8^-THC-dimethylheptyl
ICM: inner cell mass
IFN-γ: interferon-γ
IL: interleukin
IVF: *in vitro* fertilization
KO: knock-out
LCs: Leydig cells
LH: luteinizing-hormone
LIF: leukaemia inhibitory factor
LOXs: lipoxygenases
LPA: lysophosphatidic acid
LPI: l-α-lysophosphatidylinositol
lyso-NArPE: lysophosphatidylethanolamine
MAGL: monoacylglycerol lipase
MAP: mitogen-activated protein
Met-AEA: methanandamide
NADA: *N*-arachidonoyldopamine
NAE: *N*-acylethanolamine
NAPE: *N-*acylphosphatidylethanolamine
NAPE-PLD: NAPE-specific phospholipase D
N-ArPE: *N*-arachidonylphosphatidylethanolamine
NAT: *N*-acyl-transacylase
NE: norephinephrine
NFAT: nuclear factor of activated T cells
NK: natural killer
NMDA: *N*-methyl-d-aspartate
NO: nitric oxide
OVX: ovariectomized
P: progesterone
p-AEA: phospho-AEA
PC: phosphatidylcholine
PE: phosphatidyl-ethanolamine
PGE_2_: prostaglandin E_2_
PG-EA: prostaglandin ethanolamine
PG-G: prostaglandin glyceryl ester
PI: phosphatidylinositol
PKA: protein kinase A
PKC: protein kinase C
PLA_1_: phospholipase A_1_
PLC: Phospholipase C
PM: plasma membrane
PPARα: peroxisome proliferator activator receptor alpha
PRL: prolactin
R-123: rhodamine
rSPT: round spermatids
SCs: Sertoli cells
SPC: spermatocytes
SPG: spermatogonia
sPLA_2_: secretory phospholipase 2
SPT: spermatids
SPZ: spermatozoa
T: testosterone
Δ^9^-THC: Δ-9-tetrahydrocannabinol
Th: T-helper
THS: thyroid-stimulating hormone
TM: transmembrane
TRPV1: transient potential vanilloid channel type-1
URB597: cyclohexyl carbamic acid 3’-carbamoyl-byphenyl-3-yl ester
ZP: zona pellucida
